# Altered states, alkaloids, and catatonia: Monoaminoxidase inhibitors and their role in the history of psychopharmacology

**DOI:** 10.3389/fphar.2022.1053534

**Published:** 2022-12-06

**Authors:** Octavian Buda, Sorin Hostiuc, Ovidiu Popa-Velea, Steluta Boroghina

**Affiliations:** ^1^ Department of History of Medicine, Carol Davila University of Medicine and Pharmacy, Bucharest, Romania; ^2^ Legal Medicine Department, Carol Davila University of Medicine and Pharmacy, Bucharest, Romania; ^3^ Department of Medical Psychology, Carol Davila University of Medicine and Pharmacy, Bucharest, Romania

**Keywords:** psychopharmacology, catatonic schizophrenia, alkaloids, harmine, Bucharest, interwar period (1918–1938)

## Abstract

Monoamine oxidases are mitochondrial enzymes that catalyze the oxidative deamination of biogenic amines (adrenaline, noradrenaline, serotonin, and dopamine), causing their inactivation and subsequently playing a fundamental role in the homeostasis of various neurotransmitters. As the regulation of these effects was deemed important in clinical practice, numerous modulators of these enzymes were tested for various clinical effects. The purpose of this paper is to present a few historical landmarks regarding monoaminoxidase inhibitors and their usefulness as psychopharmacological agents. We will be focusing on banisterine, iproniazid, selegiline, rasagiline, tranylcypromine, moclobemide, and their role in the history of psychopharmacology. An almost unknown fact is that harmine, an MAO-A alkaloid, was used as early as the latter half of the 1920s in Bucharest, to reduce catatonic symptoms in schizophrenia, thus ushering the dawn of psychopharmacology era which started with chlorpromazine in the 1950s.

## Introduction

**FIGURE 1:**
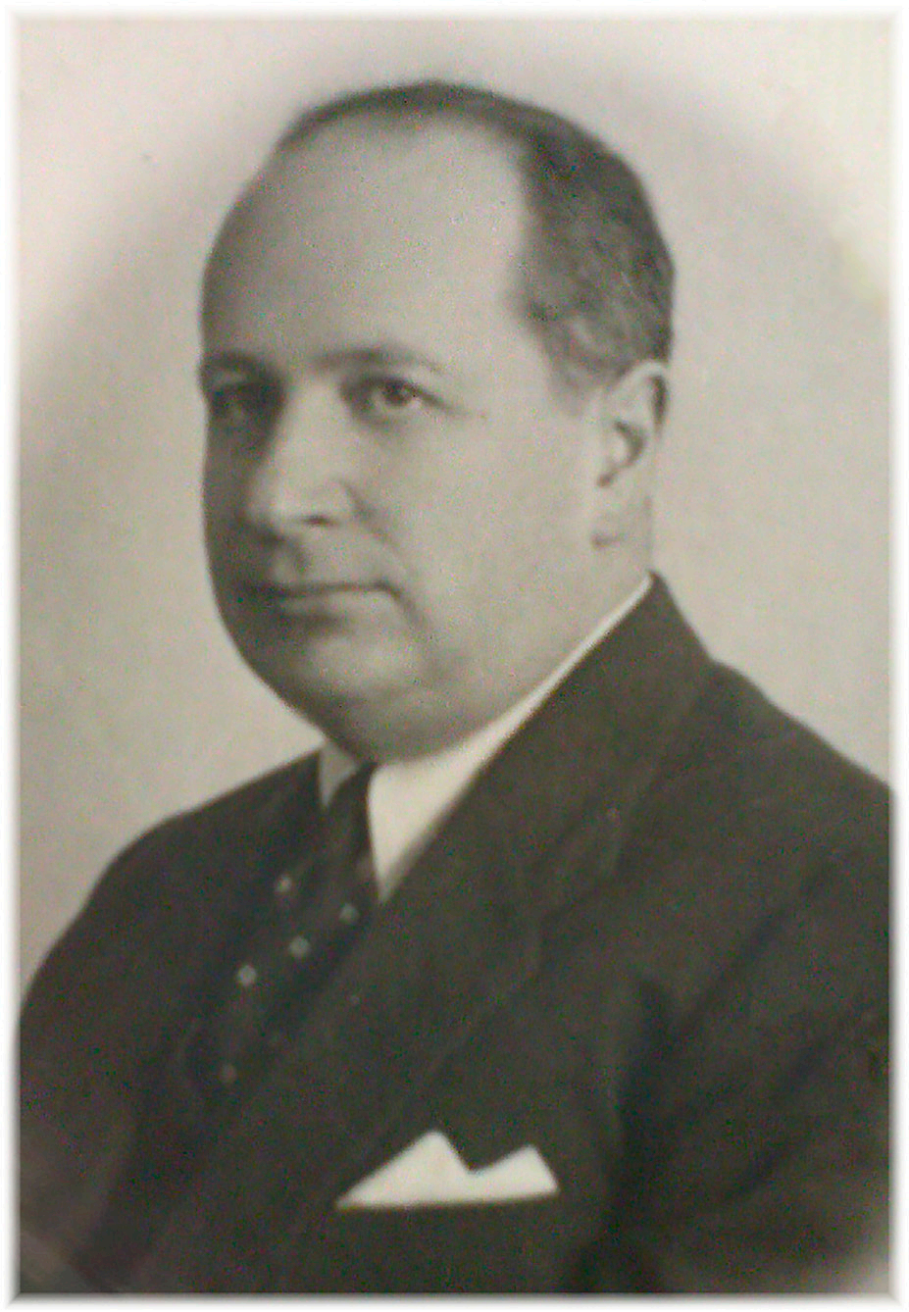
Petre Tomescu (1890–1977). Photo: Octavian Buda collection.

Soon after defining the diagnostic category of dementia praecox (Kraepelin, 1896) and schizophrenia (Bleuler, 1908), researchers and physicians tried to identify possible cures for these psychiatric diseases (Fusar-Poli and Politi, 2008; Ashok et al., 2012). Trials in this area ranged from injecting patients with animal blood (Klebelsberg, 1922) or castor oil (Ingham, 1930) to insulin therapy (Brody and Hayman, 1937; Bolles et al., 1938; Halpern, 1940; Mayer-Gross, 1951), sleep therapy (Kläsi, 1922), or even psychosurgery (Peyton et al., 1948; Feld, 1950). It was not until 1952 that researchers identified the first specific psychopharmacological treatment of schizophrenia, specifically targeted toward the elimination of psychotic symptoms (Delay et al., 1952; Lehmann and Ban, 1997). In a recent article, we described a lesser known study, published in the interwar period by a leading Romanian psychiatrist, Petre Tomescu (Figure 1),which suggested a potentially beneficial role of harmine in treating a subset of schizophrenia patients (Tomescu and Russu, 1930; Hostiuc et al., 2014; Hostiuc and Buda, 2015).

We analyze the importance of monoamine oxidase inhibitors (MAO-I) in the history of psychopharmacology and sketch a portrait of, arguably, one of the most remarkable Romanian psychiatrists, Petre Tomescu, although a more obscure personality nowadays, who introduced these pharmacological agents into the psychiatric care in Romania.

MAO are mitochondrial enzymes that catalyze the oxidative deamination of biogenic amines (adrenaline, noradrenaline, serotonin, and dopamine), determining their inactivation and subsequently playing fundamental roles in the homeostasis of various neurotransmitters (Ginovart et al., 2005). Through deamination, they release hydrogen peroxide, which is a source of hydroxyl radicals, a potentially useful target in various diseases associated with, or caused by oxidative stress. By modulating the concentration of various neurotransmitters, they were shown to be involved in addictive behaviors and personality disorders (Youdim et al., 2006). As the modulation of these effects was deemed important in clinical practice, numerous substances with effects on these enzymes were tested for various clinical effects. There are two main isoforms of MAO: MAO-A, whose inhibition was found useful for many decades in the treatment of depression and anxiety disorders (Livingston and Livingston, 1996), and MAO-B, whose inhibition was found to be beneficial for treating neurodegenerative disorders, including Parkinson’s disease (Cohen, 1990).

The first MAO-I that was widely used as a psychopharmacological agent was *Iproniazid* - isopropyl-isonicotinyl-hydrazide (Fox and Gibas, 1953), a substance initially designed as a tuberculostatic agent. The patients who had been on it showed, however, various CNS-symptoms including euphoria, psychomotor excitation, psychotic states, increased appetite, and sexual-behavior changes (Smith, 1953). This led to a clinical trial, in which institutionalized subjects diagnosed with stable depression, who received the drug, showed a significant clinical improvement in about 70% of cases just after a few weeks of treatment. The drug was subsequently introduced in clinical practice as *Marsilid®*, which is often considered the first modern antidepressant. Even if the clinical improvement was significant, Marsilid^®^ was associated with multiple severe complications including hepatic necrosis or “cheese reaction”—a hypertensive crisis in patients consuming foods containing food amines, like cheese. These severe reactions led to the withdrawal of the drug from the market (Pletscher, 1991).

## Alkaloids in neuropsychiatry

*Harmine* is another one of the many MAO inhibitors described in the literature and is a high-potency reversible inhibitor of MAO-A: IC50 = 0.0041 μM (Myburg et al., 2022). First isolated in 1847 by Carl Julius Fritzsche, *harmine* is a beta-carboline derivative, a class of plant indole alkaloids, initially obtained from the seeds of *Peganum harbala*, a plant that grows on the steppes of South Russia and in India (Gunn, 1912; Henrya Perkin, 1912). There are presently more than 60 known beta-carboline alkaloids dispersed throughout at least eight plant families, some of them hallucinogenic such as Ayahuasca and the so-called “Pharmahuasca,” which can be prepared using N,N-dimethyltryptamine or DMT (Moloudizargari et al., 2013). It acts as a competitive and reversible inhibitor of monoamine oxidase A (MAO-A). Historically, due to its chemical structure and pharmacological activity, it was hypothesized that *harmine* could be effective in treating malaria; however, the results were mediocre (Yorke and Macfie, 1924; Hill, 1929). Coulthart et al. tested the inherent bactericidal properties of various derivatives from harmine and harmaline (first isolated by Göbel in 1841), showing a peak bactericidal action of n-butyl-harmol for *B. typhosus*, n-amyl-harmol for *S. aureus*, and n-nonyl-harmol for *Amoeba* species (Coulthard et al., 1933). Throughout the 1920s, a series of clinical results were published suggesting a possible use of harmine and of a closely related substance, banisterine*,* in Parkinson’s disease (Lewin, 1928; Beringer, 1928 and Beringer, 1929; Rustige, 1929; Lewin and Schuster, 1929). For example, Rustige tested harmine, by administering it hypodermically, in doses ranging from 0.005 to 0.05 g, in 18 patients with Parkinson’s disease. In six cases, the treatment led to a clear, objective reduction of muscle rigidity, for up to 6 hours. In another nine patients, he obtained a reduction of the tremor, and in 13 patients, an improvement of voluntary movement (Rustige, 1929). Lewin described the effects of banisterine on humans and animal models. In subjects with encephalitis lethargica (cerebral flu), banisterine decreased muscle rigidity, improved the ability to walk, and had positive effects on language (Lewin, 1928). Hill tried to use harmine in patients with generalized parkinsonism, following encephalitis lethargica, but the results were mediocre, having no obvious effects, either subjective or objective, in alleviating the symptoms (Hill, 1929).

Taking into account that schizophrenic catatonia has muscle rigidity as a cardinal symptom, Petre Tomescu designed by the end of the 1920s, in Bucharest, an experiment to test whether harmine could be used as a potential treatment. The study, written in Romanian, had little to no circulation in the mainstream scientific literature, remaining almost unknown for more than 80 years (Hostiuc et al., 2014). Nowadays, catatonia is being treated with benzodiazepines, lorazepam being the first-choice drug. Electroconvulsive therapy and transcranial magnetic stimulation are second-line therapies.

After the Marsilid^®^ fiasco, MAO-I were overshadowed by more potent and less risky pharmacological alternatives. However, starting in the 1970s, various authors began to describe potential uses of these substances in schizophrenia. In 1972, it was first thought that schizophrenia subjects could have a lower MAO activity than healthy controls, but later studies deemed it unconclusive, due to the disputed reliability of the assay and ambiguous criteria for differential diagnosis between acute and chronic psychosis (Wyatt and Murphy, 1976). On the contrary, Lewine and Meltzer revealed a positive association between platelet MAO activity and negative symptoms in men with schizophrenia. Male patients diagnosed with schizophrenia and predominant negative symptoms had significantly higher MAO activity than normal controls. However, the authors also found a non-significant difference between male patients with schizophrenia and decreased negative symptoms compared with controls (Lewine and Meltzer, 1984). Recent studies have found a lower level of expression of platelet MAO in subjects with paranoid (2.37 nmol/mg protein/h), catatonic (2.29 nmol/mg protein/h), and disorganized schizophrenia (1.87 nmol/mg protein/h) than controls (4.06 nmol/mg protein/h), undifferentiated schizophrenia (4.19 nmol/mg protein/h), or manic patients (3.77 nmol/mg protein/h) (Sharma et al., 1990). Moreover, schizophrenia subjects treated with antipsychotics showed a lower platelet activity than healthy controls (Marcolin and Davis, 1992).

Furthermore, some authors have tested various MAO-I in small-scale clinical trials, suggesting potentially useful effects on subjects with schizophrenia, especially on negative symptoms. As an example, it has been shown that selegiline (an MAO-B inhibitor) is potentially useful in alleviating negative symptoms of male subjects with chronic schizophrenia, in doses between 5 and 15 mg/day (Perenyi et al., 1992). The majority of MAO inhibitors that are currently in use are irreversible inhibitors based on their mechanism of action. Selegiline (L-deprenyl, SEL) was the first one used, having been available since the late 1980s. SEL is linked with a variety of side effects, especially caused by its major L-amphetamine-like metabolite, which can cause appetite suppression, insomnia, and increased irritability (Tandaric et al., 2020).

Its propargyl analog, rasagiline (RAS), introduced a few years later, in the 1990s, features the same mechanism of action, but its metabolites have significantly less side effects, as well as a neuroprotective role. The main metabolite of RAS, 1-aminoindane, is currently being widely used, due to its proven efficacy on increasing neuron lifespan, thus allowing a lower daily dose (Azzaro et al., 2007). The effects of tranylcypromine (an unselective MAO inhibitor), when associated with chlorpromazine in chronic ambulatory subjects with schizophrenia and persistent negative symptoms, have shown to be potentially useful in preventing extra-pyramidal symptoms (Bucci, 1987). Apart from the irreversible MAO inhibitors, another useful agent is moclobemide (a reversible MAO-A inhibitor) that could improve negative, depressive, and general symptoms in subjects with schizophrenia associated with prominent negative symptoms (Silver et al., 1999).

Outside of CNS, MAO-A is preferentially expressed in the gastrointestinal tract and found in moderately higher levels in the human heart. MAO-B is preferentially expressed in the kidneys, platelets, granulocytes, and lymphocytes with a relatively equal distribution in the lungs, spleen, and liver (Belmaker et al., 1974; Wahlund et al., 1986; Ostadkarampour and Putnins, 2021). MAO-A preferentially deaminates serotonin, norepinephrine, and epinephrine and is inhibited irreversibly by low concentrations of clorgyline. MAO-B preferentially deaminates 0-phenylethylamine and benzylamine and is inhibited irreversibly by low concentrations of deprenyl (Grimsby et al., 1990).

In summary, MAOs play important roles in the metabolism of biogenic amines and regulate the concentrations of neurotransmitters in the central and peripheral nervous systems, having a major impact on the cardiac output, blood pressure, sleep, mood, cognition, and movement. The two isoforms, MAO-A and MAO-B, differ in tissue distribution, substrate selectivity, and inhibitor susceptibility. Inhibitors that act mainly on MAO-A (harmine and harmaline) are used in the treatment of depression, due to their ability to raise serotonin concentrations, while inhibitors of MAO-B decrease dopamine degradation and improve motor control in patients with Parkinson’s disease (Repic et al., 2014). Due to the widespread use of MAO inhibitors and numerous experimental and computational studies, there are several models of catalytic mechanisms on a molecular scale: the polar nucleophilic mechanism, single-electron transfer or radical mechanism, and hydride transfer mechanism (Gaweska and Fitzpatrick, 2011). MAO-B inhibition by harmanes reduces the extent of oxidative stress, and this could be a causation liaison why smokers and coffee drinkers have substantially lower incidence of neurodegeneration (Castagnoli and Murugesan, 2004). Experimental evaluations of the affinity of various types of harmanes toward MAO have been made, such as the interaction of nine types of derivatives of beta-carboline and four forms of 3,4-dihydro with purified MAO-A (Kim et al., 1997). Also, an association of MAO-A/uVNTR polymorphism has been suggested in schizophrenia, as having a predictive value. The difference in the distribution of COMT Val158Met and DAT-VNTR polymorphism supports the involvement of dopamine system components in the pathogenesis of schizophrenia (Culej et al., 2020).

## Early research on catatonia and harmine

Petre (Peter) Tomescu (born 1890, died 1977 in Bucharest) worked in his early-stage career with Alexander Obregia on psychosis and Georges Marinesco on Parkinsonism (Hostiuc et al., 2016), two of the most preeminent psychiatrists/neurologists in Romania of the first half of the 20th century. He went from being appointed lecturer in psychiatry in 1923, to an associate professor in 1927, and subsequently, professor in 1934. After 6 years, in 1940, he was appointed dean of the Faculty of Medicine of Bucharest. He was one of the first psychiatrists in Romania to be highly influenced by the genetic theories of mental pathology and eugenics, proposing a different interpretation and treatment of mental disorders compared to his predecessors. He was the only psychiatrist ever appointed as a minister of health in Romania in the 20th century, between 1941 and 1944, during the Second World War, in the far-right legionnaire government led by Ion Antonescu. In this period, his main contributions were the new Act (Law) on the Medical Infrastructure of the State in 1943 and an Act on Regulations against Sexually Transmitted Diseases (Cosmulescu, 1944). The Act on Medical Organization of the State had a highly eugenic profile, influenced by the ideas of public health physicians like Iordache Facaoaru or Iuliu Moldovan (Turda, 2009), the first objective of the act being *“to organize and direct the work of conservation and increase the prosperity of the national biological heritage”* (Act from 1943). The Act against Sexually Transmitted Diseases stated, amongst others, the fee-free treatment of these diseases and the mandatory characteristic of the prenuptial certificate (Antonescu I. gov., 1943a). He was arrested by the communist regime in 1945 and sentenced to 15 years of forced labor in 1946. Petre Tomescu was imprisoned at Jilava and Aiud, two of the most brutal labor camps for political detainees in Romania (Muraru, 2013). Subsequently, he was reinstated at the Central Hospital for Psychiatry in Bucharest (so-called Hospital No. 9) where he worked as a psychiatrist until his retirement. All his contributions, along with those of the other two preeminent psychiatrists from the interwar period (Alexander Obregia and Constantine Urechia), were suppressed by the early communist regime-imposed psychiatrists, their ideas being considered inappropriate, idealistic, and reactionary (Tudose and Tudose, 2012).

His scientific work included studies in the areas of epilepsy, physiopathology of adrenergic substances, published in 1931, psychotechnical examinations (1929 and 1930), excretion and metabolism of alcohol and other abuse substances (Tomescu and Dimolescu, 1936; Tomescu and Dimolescu, 1937; Gorun et al., 2008, Gorun et al., 2011), and psychiatric photography (Buda, 2010). One of the key areas of his studies was on catatonic syndrome, published in 1923, 1929–30. In an article from 1929, Tomescu continued work of the researchers conducted by the French neurologist Georges Bourguignon and de Jong on catatonia induced by bulbocapnine in animals by testing it on cats, dogs, chickens, and rabbits (Bourguignon, 1928; De Jong and Bourgignon, 1928). In another study, conducted together with his mentor, Al. Obregia (Buda et al., 2013), Tomescu described a series of particular cases of periodic catatonia, possible as an evolution stage of periodic psychosis, alternating with manic syndrome.

In retrospective view, his most interesting study continues to be about the potential positive effect of harmine in schizophrenic catatonia (Tomescu and Russu, 1930). The paper by Tomescu and Russu opens with a short review on the published literature by Beringer (1928) and Beringer (1929) on Parkinson’s akinesia and use of banisterine. Harmine is described as having a “pleasant intoxicating effect” by the indigenous population of South America. The authors are stating that banisterine–harmine, produced by Merck, can be used in encephalitic Parkinsonism as well and mention catatonia in connection with the opto-striate nuclei. Three patients, diagnosed explicitly with severe catatonic schizophrenia, have been subjected to harmine administration. Finding a significant positive effect on muscle rigidity, in doses between 0.01 and 0.03 cgs, the most noteworthy result of the study was a marked improvement of negative symptoms which have a slower/decreased response to conventional psychopharmacological agents. Each protocol is taking into account not only ways of administration, doses, and the improvements of catatonia but also gives a detailed psychiatric assessment of negative symptoms:

Case 1 male patient, 28 years old, diagnosed a year earlier with catatonic schizophrenia, was hospitalized on 28th of November 1928, presenting with generalized muscular rigidity, without any active movements and no ability to communicate. He was started on the following harmine protocol:

Day 1–0.01 cgs of harmine was administered *via* subcutaneous injection. No neurological effect was noted, but a lower blood pressure and heart rate were seen (14/9 mmHg to 10/7 mmHg; 74 bpm to 50 bpm).

2nd Day–0.02 cgs harmine. A slight decrease in muscle rigidity was observed.

3rd Day–0.03 cgs harmine was administered and within an hour, it was noted that the muscular rigidity disappeared in the upper limbs along with a slight improvement of the rigidity of the lower limbs and the facial muscles. Also, he started, although with difficulty, to speak being able to state facts about himself, his birth place, preferred foods, and about hospital staff. Treatment was interrupted, and over the next day, the muscular rigidity reappeared, but he maintained his ability to communicate, although he was showing signs of exhaustion. Within a week, catatonia was reinstated. Following his good initial response, he was restarted on harmine at 0.02–0.03 cgs daily for 25 days (26 January. – 21 February 1930). The patient did gain weight going from 49 to 57 kg. Due to the prohibitive price of the substance, his daily regimen was discontinued, being given occasional doses from 0.01 to 0.005 cgs, but it had been eventually stopped, moment at which, within 5–6 days, catatonia fully returned.

Case 2 male patient, 35 years old, was hospitalized on 25th of February 1927, with a catatonic schizophrenia diagnosis. In the first 2 days of treatment, he was started on 1 cgs harmine, but no effect was seen. In the following 3 days, the doses had been increased to 2 cgs, 3 cgs, and 4 cgs, respectively. Subsequently, the muscular rigidity had completely disappeared, facial mimicry became adequate, was able to feed himself, communicate verbally, and presented interest to interact with the medical personnel. Even though the catatonia was not as intense as in the first case, the positive effects of harmine lasted around 2–3 days after its interruption, and subsequently, catatonia was reinstated 20 days later.

Case 3 male patient, 32 years old, diagnosed with severe catatonic schizophrenia, was hospitalized on 27th of January 1925. The patient had not had any interaction with medical staff from the summer of 1927 until January 1930. He was started during the first 4 days on 0.02 cgs harmine, with an increase in dose on the fifth day by 0.01 cgs. Following the increase in dose, a rapid clinical improvement was seen, with the patient starting to communicate verbally, having a good appetite, and presenting insight by stating that earlier he did not want to talk because he felt extremely sick. Also, he expressed the willingness to go home and reunite with his family, suggesting that his emotional status was excellent. After 20 days, the treatment was discontinued, and within 5 days, the intense negative symptoms were back.

**FIGURE 2:**
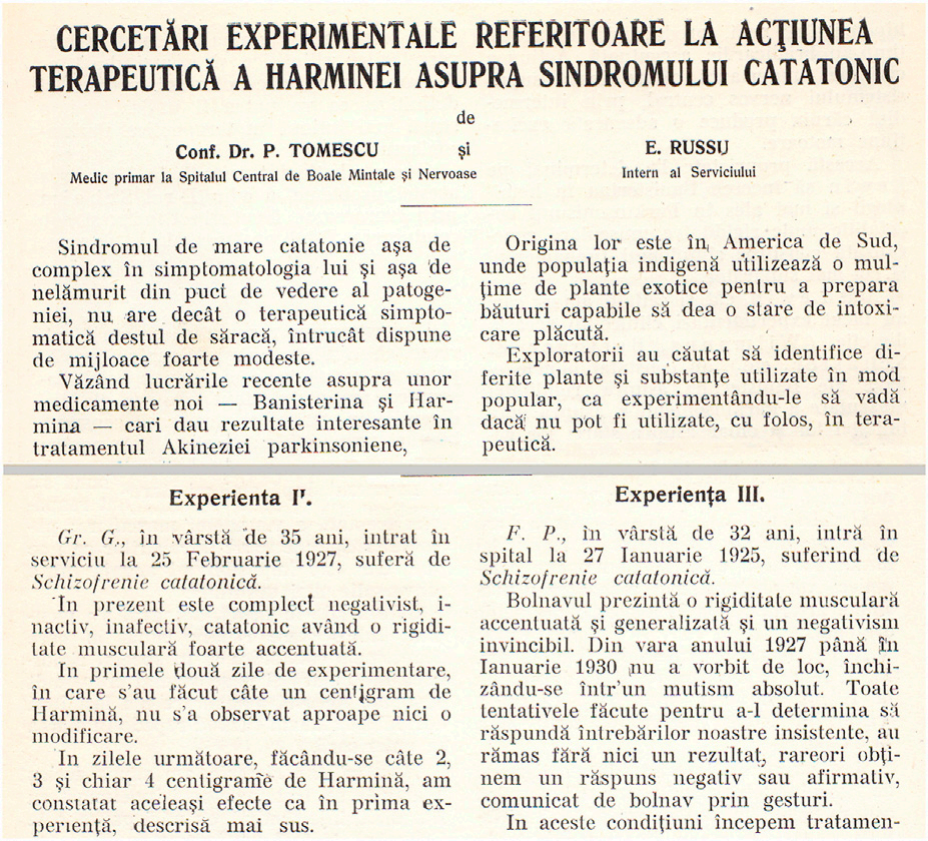
Tomescu and Russu’s paper on the use of harmine in catatonic syndrome, 1930, courtesy of ‘Mina Minovici’ Institute Library, Bucharest.

Tomescu and Russu used harmine in schizophrenia and noticed that alongside catatonia, it was also improving negative symptoms, thus making it a psychopharmacologic substance (MAO-A), long before the first use of chlorpromazine (thorazine) in 1952 by Henri Laborit et al.(Healy, 2004). The effect, even though temporary, was profound, especially on negative symptoms, and furthermore, recent studies should have been performed to analyze its potential effectiveness. He was not able to continue these studies, especially due to the prohibitive costs of harmine at that time (Figure 2).

Dr. Tomescu is reminiscent of the movie character of a neurologist, Dr. Malcolm Sayer (actually Oliver Sacks), played by Robin Williams, who administered L-dopa to catatonic patients who survived the 1917–1928 epidemic of encephalitis lethargica (Awakenings, directed by Penny Marshall, 1990). One of the patients, Leonard Lowe (a character played by Robert de Niro), woke after decades and had to deal with a new life but sadly for a short time as he returned to his previous catatonic state (Sacks, 1973).
